# Development of an Epoxy-Based Rapid Tool with Low Vulcanization Energy Consumption Channels for Liquid Silicone Rubber Injection Molding

**DOI:** 10.3390/polym14214534

**Published:** 2022-10-26

**Authors:** Chil-Chyuan Kuo, Qing-Zhou Tasi, Song-Hua Hunag

**Affiliations:** 1 Department of Mechanical Engineering, Ming Chi University of Technology, New Taipei City 243, Taiwan; 2 Research Center for Intelligent Medical Devices, Ming Chi University of Technology, No. 84, Gungjuan Road, New Taipei City 243, Taiwan; 3 Li-Yin Technology Co., Ltd., No. 37, Lane 151, Section 1, Zhongxing Road, Wugu District, New Taipei City 241, Taiwan

**Keywords:** liquid silicone rubber, conformal heating channel, conformal cooling channel, vulcanization, energy consumption

## Abstract

Liquid silicone rubber (LSR) parts have some distinct characteristics such as superior heat stability, low-temperature flexibility, aging resistance, and chemical resistance. From an industrial standpoint, the uniform vulcanization temperature of LSR is an important research point. However, the uniformity of the vulcanization temperature of LSR has been limited since the layout of the cartridge heater incorporated in the conventional steel mold does not follow the profile of the mold cavity. Metal additive manufacturing can be used to make LSR injection molds with conformal heating channels and conformal cooling channels simultaneously. However, this method is not suitable for a mold required to develop a new LSR product. In this study, a cost-effective approach was proposed to manufacture an LSR injection mold for the pilot run of a new optical lens. A rapid tool with low vulcanization energy consumption channels was proposed, which was incorporated with both a conformal heating channel (CHC) and conformal cooling channel (CCC) simultaneously. The function of the CHC was to vulcanize the LSR in the cavity uniformly, resulting in a shorter cycle time. The function of the CCC was to keep the LSR in a liquid state for reducing runner waste. It was found that the equation of y = −0.006x^3^ + 1.2114x^2^ − 83.221x + 1998.2 with the correlation coefficient of 0.9883 seemed to be an optimum trend equation for predicting the solidification time of a convex lens (y) using the vulcanizing hot water temperature (x). Additionally, the equation of y = −0.002x^3^ + 0.1329x^2^ − 1.0857x + 25.4 with the correlation coefficient of 0.9997 seemed to be an optimum prediction equation for the solidification time of a convex lens (y) using the LSR weight (x) since it had the highest correlation coefficient. The solidification time of a convex lens could be reduced by about 28% when a vulcanizing hot water temperature of 70 °C was used in the LSR injection mold with CHC.

## 1. Introduction

Liquid silicone rubber (LSR) injection molding is a promising method for the mass production of parts with sophisticated geometries because of its ease of processability [1]. The injection molding of LSR is ideal for rubber parts in specific demands, such as in the medical, automotive, aerospace, electrical, and consumer industries since it provides better end-product performance [2]. It was found that the thermal stability of addition-cured liquid silicone rubber was improved significantly. A conformal cooling channel (CCC) was employed in the plastic injection molding to enhance productivity and molded part quality [3,4,5]. 

Generally, the more uniform the mold temperature, the shorter the cross-linking time of LSR. According to practical experience in the fabrication of LSR parts using injection molding [6,7], one major drawback is that the uniformity of the vulcanization temperature of LSR can be improved when a cartridge heater [8] is incorporated in the conventional mold steel. Thus, improving the uniformity of the vulcanization temperature of LSR is an important research issue. A conformal heating channel (CHC) and a CCC can be realized simultaneously by metal additive manufacturing (AM). However, there are many distinct processing defects, including warpage [9], residual stress [10], and cracking [11]. In addition, hitherto little has been reported on the use of a CHC and CCC simultaneously in LSR injection molds. To overcome this challenge, a rapid tool with dual channels was proposed and implemented, which was incorporated with conformal heating and cooling channels simultaneously in this study. A CHC is a heating passageway, which follows the shape of the mold cavity, providing higher uniformity of the vulcanization temperature of LSR after injection molding. A CCC is used to maintain the LSR in a liquid state in the filling system. Conventionally, both CHC and CCC have been typically designed by a trial-and-error method in which multiple attempts are made to reach a solution. However, this is a time-consuming method. In this study, numerical simulation was used as an effective way for designing both the CHC and CCC. The feasibility of the LSR injection mold with dual channels was fabricated with aluminum (Al)-filled epoxy resin. To validate the simulation results and evaluate the effectiveness of the LSR injection mold, LSR injection molding was carried out. Thermal imaging technology can help to characterize the changes in the vulcanization process. Thus, an infrared thermal imager was also employed to record the temperature history during LSR injection molding.

## 2. Experimental Details

**Figure 1:**
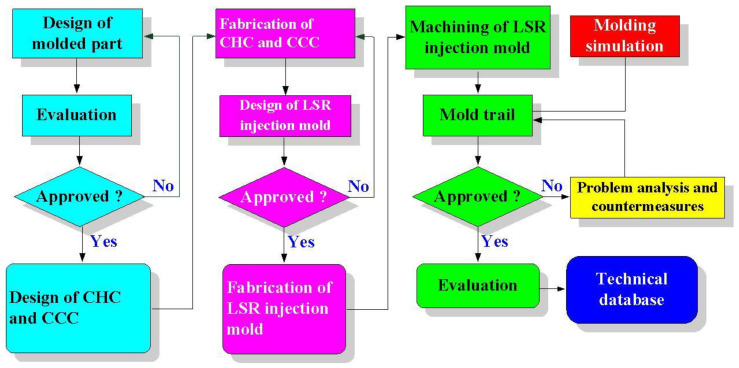
Flow diagram of the experimental methodology.

**Figure 2:**
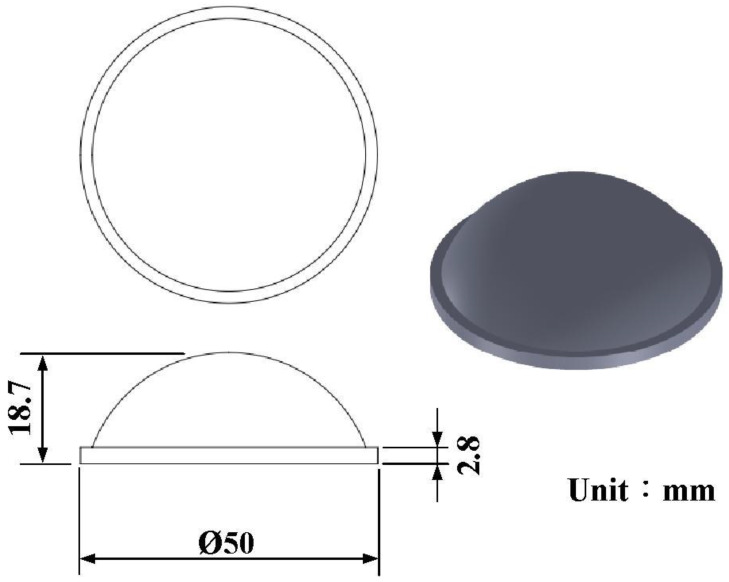
Three-dimensional CAD model and dimensions of a convex lens.

**Figure 3:**
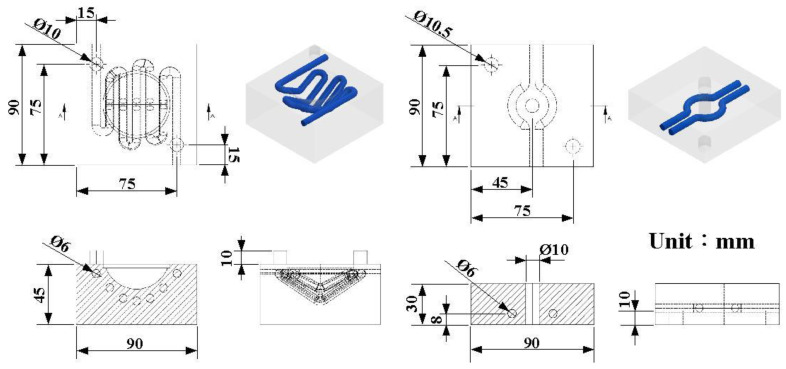
Three-dimensional CAD model and dimensions of cavity with CHC and core with CCC.

In this study, a convex lens of a vehicle headlight was selected as the master pattern. The master pattern, CHC, CCC, and LSR injection molds were designed using a three-dimensional (3D) modeling software (Parametric Technology Corporation, Boston, MA, USA). Figure 1 shows the flow diagram of the experimental methodology. Figure 2 shows the 3D CAD model and dimensions of the convex lens. The diameter of the convex lens of the vehicle headlight was about 50 mm and thickness of the center was about 18.7 mm. Figure 3 shows the 3D CAD model and dimensions of the cavity with the CHC and core with the CCC. CHCs achieve a better vulcanization performance than conventional heating channels. In addition, CCCs achieve better cooling performance than conventional straight-drilled channels. The core insert had a length of 90 mm, a width of 90 mm, and a height of 30 mm. The cavity insert had a length of 90 mm, a width of 90 mm, a height of 45 mm. The diameter of the CHC was 6 mm. The pitch distance between the central lines of the CHC was 10 mm. The distance between the wall of the CHC to the mold surface was 8 mm. The diameter of the CCC was 6 mm. The distance between the wall of the CCC to the mold surface was 8 mm. A set of LSR injection molds with dual CCCs was fabricated using Al-filled epoxy resins (TE-375, Jasdi Inc., New Taipei City, Taiwan).

**Figure 4:**
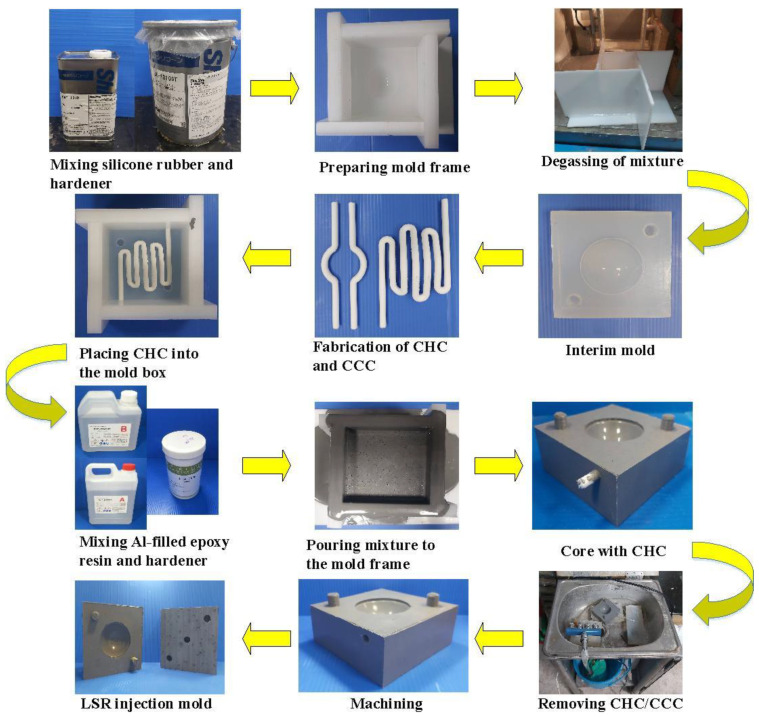
Manufacturing processes of LSR injection mold.

**Figure 5:**
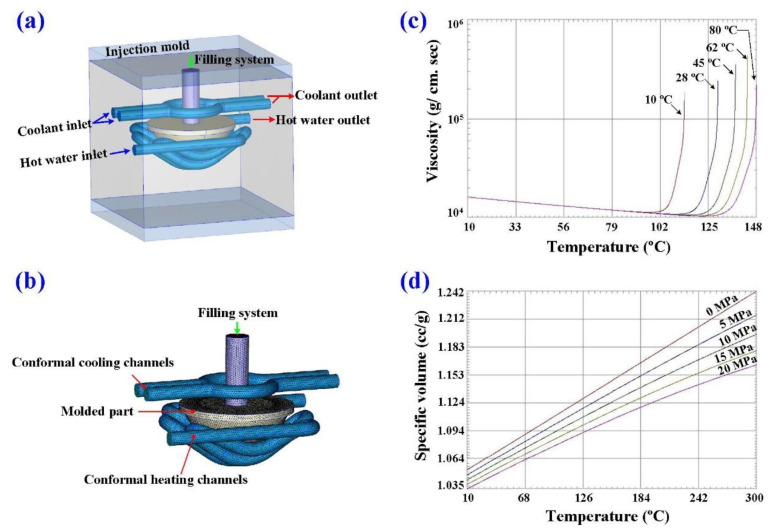
Description of molding simulation conditions: (**a**) configuration of the injection mold, (**b**) finite-element mesh, (**c**) viscosity chart, and (**d**) PVT diagram of the molding material.

Figure 4 shows the manufacturing processes of the LSR injection mold. Firstly, an interim mold, which was complementary in shape to the LSR injection mold, was fabricated by silicone rubber (KE-1310ST, Shin Etsu Inc., Chiyoda City, Japan) and a hardener. The hardener and silicone rubber were mixed in a weight ratio of 1:10. To reduce human error, a computer program using Visual Basic was developed to determine the amounts of both the base compound and hardener accurately. The mixture was blended with epoxy resins (EP-2N1, Ruixin Inc., Taipei, Taiwan) and 41 vol.% aluminum (Al) powder [12] for manufacturing the LSR injection mold. The average particle size of Al powder was about 45 µm. The mixture was stirred for about 15–20 min until the mixture was well blended. The mixture was then de-gassed by a vacuum pump (F-600, Feiling Inc., New Taipei City, Taiwan). Both the core and cavity inserts were then post-cured in a thermal oven at 60 °C to obtain the mechanical properties. The fabrication of CHCs using conventional machining techniques is difficult. Thanks to the features of three-dimensional printing technology [13], both the CHC and CCC were printed using polyvinyl butyral [14] filament because these materials can be removed thoroughly and easily by using industrial alcohol solution [15]. The printing parameters for manufacturing both the CHC and CCC were a printing speed [16] of 30 mm/s, printing temperature [17] of 200 °C, layer thickness [18] of 0.1 mm, and bed temperature [19] of 60 °C. Figure 5 shows the description of the molding simulation conditions. The viscosity is the index of the resistance of an LSR to flow, which depends on temperature, shear rate, and pressure. In general, LSR undergoes a significant volumetric change with respect to pressure and temperature. To calculate the shrinkage or warpage of a convex lens after vulcanization, the characterization of the pressure–volume–temperature (PVT) relationship was required.

**Figure 6:**
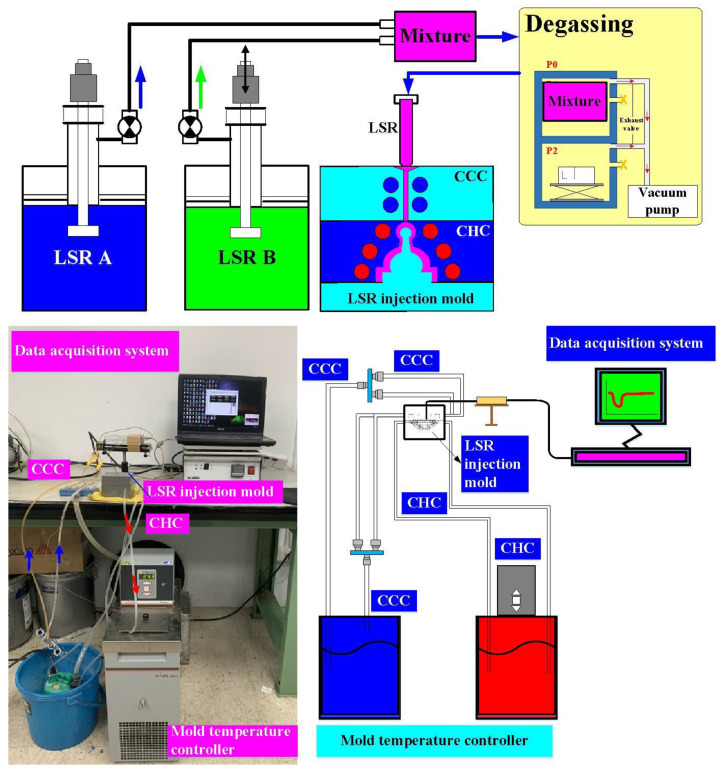
A rapid tool with CHC and CCC for LSR injection molding.

The Moldex3D simulation software (R14 SP3OR, CoreTech System Inc., Zhubei City, Taiwan) is an effective tool for the design verification of both CHCs and CCCs. Table 1 gives the process conditions used in the simulation. The filling time of a convex lens was approximately 1 s. Table 2 shows the material properties of the injection mold. LSR (ELASTOSIL LR 3003 50, Wacker Inc., Munich, Germany) was used as the molding materials and the material properties are listed in the Table 3. Figure 6 shows a rapid tool with a CHC and CCC for LSR injection molding. The cross-section of both the CHC and CCC was circular. A vacuolization and cooling system for LSR injection molding was developed in this study. This system comprised a CHC, CCC, three k-type thermocouples (C071009-079, Cheng Tay Inc., Taipei, Taiwan) with a measurement sensitivity of ±1 °C, a mold temperature controller (JCM-33A, Shinko Inc., Taipei, Taiwan), a water reservoir with a thermoelectric cooler (TEC12706AJ, Caijia Inc., Taipei, Taiwan), and a data acquisition system (MRD-8002L, IDEA System Inc., Taipei, Taiwan). To investigate the effect of the hot-water temperature on the vulcanizing results, five different vulcanizing hot-water temperatures, i.e., 50 °C, 55 °C, 60 °C, 65 °C, and 70 °C, were used in this study. The cold-water temperature was 27 °C with a volume flow rate of 120 cc/s. The temperature of the molded part after molding was recorded by an infrared camera (BI-TM-F01P, Panrico Trading Inc., Taipei, Taiwan).

## 3. Results and Discussion

**Figure 7:**
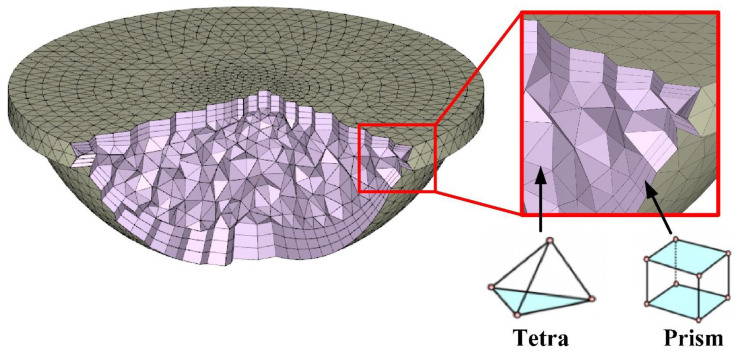
Mesh of the injection molded part.

In this study, a boundary layer mesh was employed for the simulation because it is suitable for simulation models with sophisticated geometries. A three-dimensional solid mesh includes four different kinds of meshes, i.e., prism, tetrahedron (tetra), pyramid, and hexahedron. Figure 7 shows the mesh of the injection molded par. It should be noted that the simulation models were composed of meshes with tetrahedrons and prisms. The number of nodes for tetrahedra and prisms were 4 and 8 [20]. The number of elements for tetrahedra and prisms were 19,768 and 10,740, respectively. The simulation models involved a molded part, a mold base, two channels, and a runner. The total number of elements and nodes were 30,508, 145,474, 1,293,268, and 12,989, respectively. 

**Figure 8:**
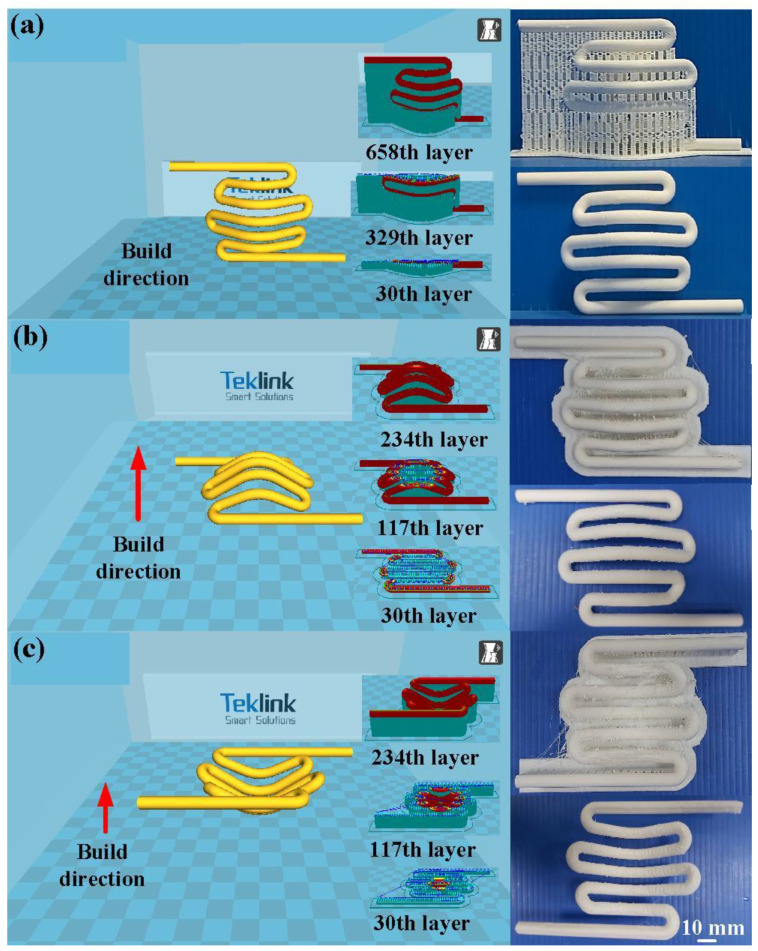
CHC before and after removing support materials using three different build directions: (**a**) vertical, (**b**) horizontal, and (**c**) inverted.

Figure 8 shows the CHC before and after removing the support materials using three different build directions. The printing time of the CHC for vertical, horizontal, and inverted directions were 380, 300, and 396 min, respectively. It should be noted that serious warpage of CHC was observed after removing the support materials while the build direction was vertical. Especially, there were many support materials when the CHC was built with inverted direction that resulted in more time taken to remove support material. It is interesting to conclude that the optimal build direction was horizontal.

**Figure 9:**
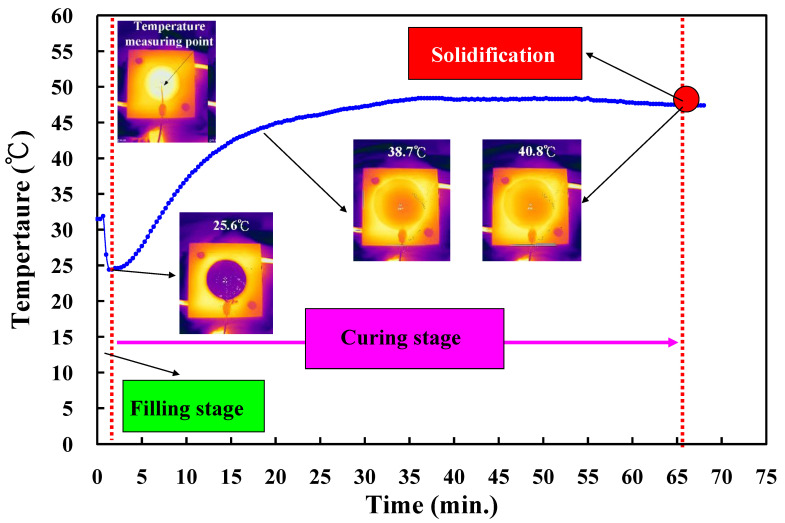
Temperature of a convex lens as a function of vulcanization time.

The curing process is based on chemical reaction kinetics. The vulcanization time of an LSR has a significant impact on molding product of a convex lens because the vulcanization is a crucial step in LSR injection molding [21,22,23]. Generally, both CHCs and CCCs are a complex heat transfer problem with transient conditions. To prevent the over-curing of the LSR during LSR injection molding [6,7], five curing temperatures were used in this study. First of all, a vulcanizing hot-water temperature of 60 °C was employed to investigate the curing time [24,25] of the molding product. Figure 9 shows the temperature of a convex lens as a function of the vulcanization time. The temperature history during the vulcanization of the LSR was monitored by an infrared thermal imager in the LSR injection molding. Note that there was only one temperature measuring point, which was located in the center of the bottom of the optical lens. The curing rate of an LSR lens is related to heat released by the curing reaction. The curing rate of the LSR lens was predicted by molding simulation. The performance of the LSR lens was affected by the mechanism of curing kinetics since curing is an exothermic process of crosslinking. Since the temperature sensor was placed on the top of the molded part, the temperature of the molded part first decreased and then increased with the increase in the vulcanization time. According to the temperature of the infrared thermal imager, the peak temperature of the top of the silicone optical lens was about 45 °C. According to both the temperature of the molded part and vulcanization time, the solidification time of a convex lens was about 66 min.

**Figure 10:**
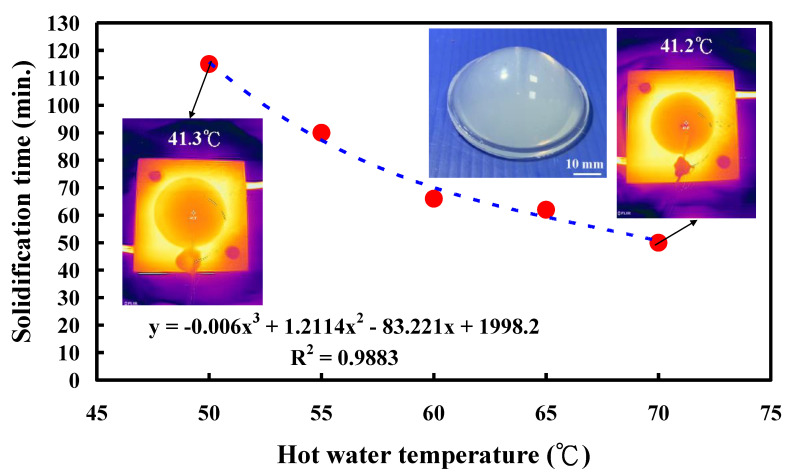
Solidification time of a convex lens for different vulcanizing hot-water temperatures.

In practice, little is known about the relationship between the vulcanizing hot-water temperature and the solidification time of a convex lens. To investigate the relationship between the vulcanizing hot-water temperature and the solidification time of a convex lens, five different vulcanizing hot-water temperatures of 50 °C, 55 °C, 60 °C, 65 °C, and 70 °C were performed in this study. Figure 10 shows the solidification time of a convex lens for different vulcanizing hot-water temperatures. The solidification times of the convex lenses were approximately 115, 90, 66, 62, and 50, respectively. To propose an optimum trend equation for predicting the solidification time of a convex lens based on the vulcanizing hot-water temperature, six different types of curve-fitting methods were used in this study, as shown in the following Equations (1) to (6). Note that the R^2^ stands for the coefficient of determination of the trend equation. In general, the R^2^ value ranges from 0 to 1 and is commonly stated as a percentage from 0% to 100%. The higher the R^2^ value, the better the degree of accuracy of the equation. The values of x and y denote the vulcanizing hot-water temperature and solidification time of a convex lens, respectively. Apparently, it was found that the equation of y = −0.006x^3^ + 1.2114x^2^ − 83.221x + 1998.2 was considered to be the optimum trend equation for predicting the solidification time of a convex lens (y) using vulcanizing hot-water temperature (x) since it had the highest R^2^ value.

Linear function:

y = −3.16x + 266.2, R^2^ = 0.929 (1)

Quadratic function:

y = 0.1314x^2^ − 18.931x + 732.77, R^2^ = 0.9852 (2)

Exponential function:

y = 846.32e^−0.0408x^, R^2^ = 0.9656 (3)

Logarithmic function:

y = −189.95 ln(x) + 852.99, R^2^ = 0.9498 (4)

Cubic function:

y = y = −0.006x^3^ + 1.2114x^2^ − 83.221x + 1998.2, R^2^ = 0.9883 (5)

Power function:

y = 2 × 10^6^x^−2.4364^, R^2^ = 0.9778 (6)

**Figure 11:**
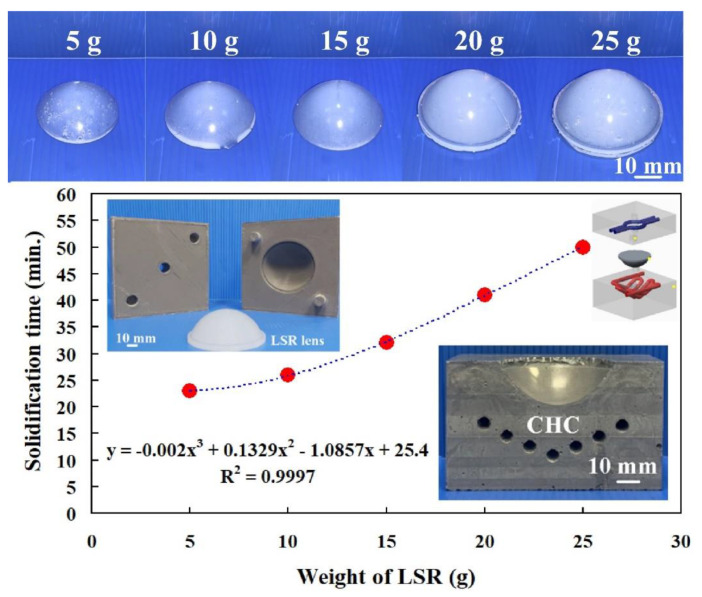
Solidification time of a convex lens for different LSR weights.

The weight of the complete LSR convex lens was 25 g. To investigate the relationship between the LSR volume and the solidification time of a convex lens, five different LSR volumes of 20%, 40%, 60%, 80%, and 100% were carried out in this study. Five different weights of the LSR, i.e., 5 g, 10 g, 15 g, 20 g, and 25 g were obtained by converting five different volumes of the LSR. Figure 11 shows the solidification time of a convex lens for different LSR weights. To propose an optimum trend equation for predicting the solidification time of a convex lens based on the LSR weight, six different kinds of functions were used in this study, as shown in the following Equations (7) to (12). The values of x and y denote the LSR weight and the solidification time of a convex lens, respectively. It is worth noting that it was found that the equation of y = −0.002x^3^ + 0.1329x^2^ − 1.0857x + 25.4 seemed to be the optimum prediction equation for the solidification time of a convex lens (y) using the LSR weight (x) since it had a highest correlation coefficient.

Linear function:

y = 1.38x + 13.7, R^2^ = 0.9653 (7)

Quadratic function:

y = 0.0429x^2^ + 0.0943x + 21.2, R^2^ = 0.9979 (8)

Exponential function:

y = 18.073e^0.0402x^, R^2^ = 0.9891 (9)

Logarithmic function:

y = 15.969 ln(x) − 6.5913, R^2^ = 0.8353 (10)

Cubic function:

y = −0.002x^3^ + 0.1329x^2^ − 1.0857x + 25.4, R^2^ = 0.9997 (11)

Power function:

y = 9.7392x^0.4756^, R^2^ = 0.8959 (12)

**Figure 12:**
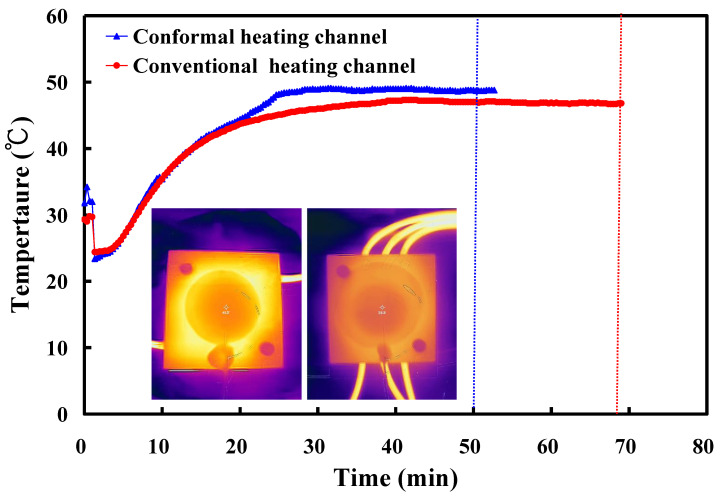
Solidification time of a convex lens for LSR injection mold with CHC and conventional heating channel.

**Figure 13:**
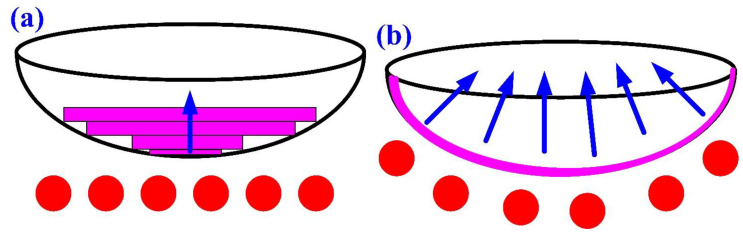
Vulcanization mechanism of a convex lens fabricated by LSR injection mold with (**a**) conventional heating channel and (**b**) CHC.

**Figure 14:**
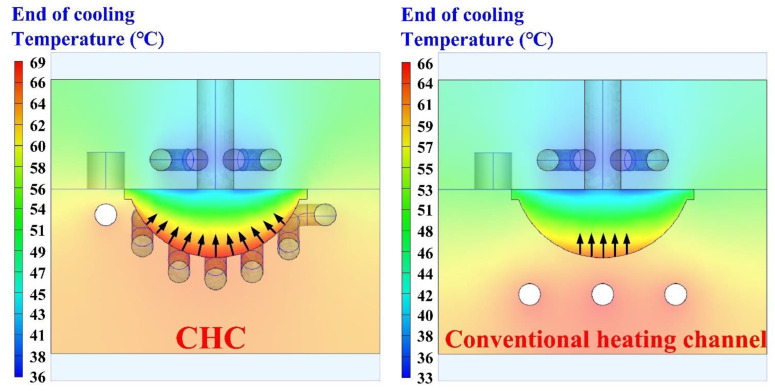
Numerical simulation results of a convex lens fabricated by LSR injection mold with CHC and conventional heating channel.

To investigate the effectiveness of the CHC in the solidification time of a convex lens, a vulcanizing hot-water temperature of 70 °C was used in the experiment. Figure 12 shows the solidification time of a convex lens for the LSR injection mold with a CHC and a conventional heating channel. As can be seen, the surface temperature of a convex lens using the CHC was about 3 °C higher than that of the conventional heating channel. This result showed that the heat transfer performance of the CHC was better than that of the traditional heating channel. It should be noted that two phenomena were found. One was that the solidification times of a convex lens for the LSR injection mold with a CHC and a conventional heating channel were 50 min and 69 min, respectively. The other was that a saving of the solidification time of a convex lens of about 28% could be obtained when using an LSR injection mold with a CHC. Figure 13 shows the vulcanization mechanism of a convex lens fabricated by an LSR injection mold with a conventional heating channel and a CHC. Note that the arrows indicate the curing direction of the LSR. It should be noted that the curing sequence of a convex lens was from the exterior of the convex lens to the interior of the convex lens when a CHC was used in the LSR injection mold. This means that the vulcanization uniformity of a convex lens was better, and the solidification time of a convex lens was shorter when a CHC was used in the LSR injection mold. However, the curing sequence of a convex lens was from the bottom of the convex lens to the top of the convex lens when a conventional heating channel was used in the LSR injection mold. Thus, the LSR injection mold with a CHC had a tremendous impact on the productivity of mass production compared to the LSR injection mold with a conventional heating channel. Figure 14 shows the numerical simulation results of a convex lens fabricated by an LSR injection mold with a CHC and a conventional heating channel. The results showed that the numerical simulation results were in good agreement with the experimental results.

In this study, a sustainable LSR injection mold with both conformal heating and conformal cooling channels was demonstrated. This injection mold was a green mold [26] and met sustainable development goals [27]. Therefore, the findings of this study are very practical and provide the greatest application potential in the design stage of an LSR injection mold. Unfortunately, both the mechanical and physical properties of the LSR injection mold fabricated with Al-filled epoxy resins were not better than those fabricated from a conventional steel mold. Thus, enhancing both the mechanical and physical properties of an LSR injection mold by adding some different kinds of reinforcing fillers, such as carbon fibers [28], zirconia particles [29], silicon nitride particles [30], or molybdenum disulfide particles [31] in Al-filled epoxy resin is also an important research topic. The optimization of the mechanical and physical properties using the Taguchi method for an LSR injection mold could also be investigated [32]. Additionally, the optimization of the curing phase of an LSR injection molding using the Moldex 3D simulation software is also an important research issue [33,34,35]. These issues are currently being investigated and the results will be presented in a later study.

## 4. Conclusions

An LSR possesses some distinct properties, such as chemical stability, high temperature resistance, flame resistance, corrosion resistance, and electrical insulation. However, hitherto little has been reported on the use of a CHC and CCC simultaneously in an LSR injection mold. In this study, an energy-saving LSR injection mold with conformal heating and conformal cooling hybrid channels was implemented. The effects of both the vulcanizing hot-water temperature and the LSR volume on the solidification time of a convex lens were investigated experimentally. The main conclusions from the experimental work in this study are as follows:

1. The remarkable findings in this study were very practical and provide potential applications in the LSR injection molding industry because an injection mold with both a CHC and CCC for LSR injection molding was possible.
2. The equation of y = −0.006x^3^ + 1.2114x^2^ − 83.221x + 1998.2 with a correlation coefficient of 0.9883 was the optimum trend equation for predicting the solidification time of a convex lens (y) using the vulcanizing hot-water temperature (x).
3. The equation of y = −0.002x^3^ + 0.1329x^2^ − 1.0857x + 25.4 with a correlation coefficient of 0.9997 was the optimum prediction equation for the solidification time of a convex lens (y) using the LSR weight (x).
4. A saving in the solidification time of a convex lens of about 28% could be obtained when a vulcanizing hot-water temperature of 70 °C was employed in the LSR injection mold with a CHC.

## Figures and Tables

**Table 1:** Process conditions used in the simulation.

Item	Data
Injection temperature (°C)	27
Hot-water temperature (°C)	50, 55, 60, 65, 70
Coolant temperature (°C)	27
Flow rate (cc/s)	120
Injection pressure (MPa)	0.52
Filling time (s)	1

**Table 2:** Material properties of injection mold.

Item	Data
Density (g/cm^3^)	1.95
Heat capacity (cal/g °C)	1.97
Thermal conductivity (W/m-K)	10.82
Elastic modulus (GPa)	2.54
Poisson ratio	0.17

**Table 3:** Properties of the molding materials.

Item	Data
Density (g/cm^3^)	1.04
Hardness (Shore A)	60
Material temperature (°C)	10–30

## Data Availability

Not applicable.

## References

[1] Guo J., Wang X., Wang J., Chen C., Liu Y., Fan W., Jia Z. (2019). Study on the Anticondensation Characteristics of Liquid Silicone Rubber Temperature-Control Coatings. Polymers.

[2] Woitschach F., Kloss M., Schlodder K., Borck A., Grabow N., Reisinger E.C., Sombetzki M. (2021). In Vitro Study of the Interaction of Innate Immune Cells with Liquid Silicone Rubber Coated with Zwitterionic Methyl Methacrylate and Thermoplastic Polyurethanes. Materials.

[3] Mohd Hanid M.H., Abd Rahim S.Z., Gondro J., Sharif S., Al Bakri Abdullah M.M., Zain A.M., El-hadj Abdellah A., Mat Saad M.N., Wysłocki J.J., Nabiałek M. (2021). Warpage Optimisation on the Moulded Part with Straight Drilled and Conformal Cooling Channels Using Response Surface Methodology (RSM), Glowworm Swarm Optimisation (GSO) and Genetic Algorithm (GA) Optimisation Approaches. Materials.

[4] Oh S.-H., Ha J.-W., Park K. (2022). Adaptive Conformal Cooling of Injection Molds Using Additively Manufactured TPMS Structures. Polymers.

[5] Kuo C.-C., Chen W.-H. (2021). Improving Cooling Performance of Injection Molding Tool with Conformal Cooling Channel by Adding Hybrid Fillers. Polymers.

[6] Kuo C.C., Lin J.X. (2019). A cost-effective method for rapid manufacturing polymer rapid tools used for liquid silicone rubber injection molding. Int. J. Adv. Manuf. Technol..

[7] Kuo C.C., Lin J.X. (2019). Fabrication of the Fresnel lens with liquid silicone rubber using rapid injection mold. Int. J. Adv. Manuf. Technol..

[8] Shu Y., Chen T., Zhou W., Zhou Z., Yi A.Y. (2021). Fabrication of large-scale infrared diffractive lens arrays on chalcogenide glass by means of step-and-repeat hot imprinting and non-isothermal glass molding. Int. J. Adv. Manuf. Technol..

[9] Marin F., de Souza A.F., Ahrens C.H., de Lacalle L.N.L. (2021). A new hybrid process combining machining and selective laser melting to manufacture an advanced concept of conformal cooling channels for plastic injection molds. Int. J. Adv. Manuf. Technol..

[10] Xiaohui J., Chunbo Y., Honglan G., Shan G., Yong Z. (2022). Effect of supporting structure design on residual stresses in selective laser melting of AlSi10Mg. Int. J. Adv. Manuf. Technol..

[11] Pekok M.A., Setchi R., Ryan M., Han Q., Gu D. (2021). Effect of process parameters on the microstructure and mechanical properties of AA2024 fabricated using selective laser melting. Int. J. Adv. Manuf. Technol..

[12] Kuo C.-C., Xu J.-Y., Zhu Y.-J., Lee C.-H. (2022). Effects of Different Mold Materials and Coolant Media on the Cooling Performance of Epoxy-Based Injection Molds. Polymers.

[13] Cuan-Urquizo E., Álvarez-Trejo A., Robles Gil A., Tejada-Ortigoza V., Camposeco-Negrete C., Uribe-Lam E., Treviño-Quintanilla C.D. (2022). Effective Stiffness of Fused Deposition Modeling Infill Lattice Patterns Made of PLA-Wood Material. Polymers.

[14] Channa I.A., Chandio A.D., Rizwan M., Shah A.A., Bhatti J., Shah A.K., Hussain F., Shar M.A., AlHazaa A. (2021). Solution Processed PVB/Mica Flake Coatings for the Encapsulation of Organic Solar Cells. Materials.

[15] Ari B., Sahiner M., Demirci S., Sahiner N. (2022). Poly(vinyl alcohol)-tannic Acid Cryogel Matrix as Antioxidant and Antibacterial Material. Polymers.

[16] Weiss L., Sonsalla T. (2022). Investigations of Fused Deposition Modeling for Perovskite Active Solar Cells. Polymers.

[17] Hu J., Mubarak S., Li K., Huang X., Huang W., Zhuo D., Li Y., Wu L., Wang J. (2022). The Micro–Macro Interlaminar Properties of Continuous Carbon Fiber-Reinforced Polyphenylene Sulfide Laminates Made by Thermocompression to Simulate the Consolidation Process in FDM. Polymers.

[18] Rijckaert S., Daelemans L., Cardon L., Boone M., Van Paepegem W., De Clerck K. (2022). Continuous Fiber-Reinforced Aramid/PETG 3D-Printed Composites with High Fiber Loading through Fused Filament Fabrication. Polymers.

[19] Mader M., Rein C., Konrat E., Meermeyer S.L., Lee-Thedieck C., Kotz-Helmer F., Rapp B.E. (2021). Fused Deposition Modeling of Microfluidic Chips in Transparent Polystyrene. Micromachines.

[20] Kuo C.C., Xu Y.X. (2022). A simple method of improving warpage and cooling time of injection molded parts simultaneously. Int. J. Adv. Manuf. Technol..

[21] Bex G.-J., Desplentere F., De Keyzer J., Van Bael A. (2017). Two-component injection moulding of thermoset rubber in combination with thermoplastics by thermally separated mould cavities and rapid heat cycling. Int. J. Adv. Manuf. Technol..

[22] Ou H., Sahli M., Gelin J.C., Barrière T. (2017). Multiphysics modelling and experimental investigations of the filling and curing phases of bi-injection moulding of thermoplastic polymer/liquid silicone rubbers. Int. J. Adv. Manuf. Technol..

[23] Ou H., Sahli M., Barriere T., Gelin J.C. (2017). Experimental characterisation and modelling of rheokinetic properties of different silicone elastomers. Int. J. Adv. Manuf. Technol..

[24] Roth B., Drummer D. (2021). Pressure Equilibrium Time of a Cyclic-Olefin Copolymer. Polymers.

[25] Roth B., Wildner W., Drummer D. (2020). Dynamic Compression Induced Solidification. Polymers.

[26] Khan K., Gudainiyan J., Iqbal M., Jamal A., Amin M.N., Mohammed I., Al-Faiad M.A., Abu-Arab A.M. (2022). Modelling Compression Strength of Waste PET and SCM Blended Cementitious Grout Using Hybrid of LSSVM Models. Materials.

[27] Kumar L., Jain P.K., Sharma A.K. (2020). A fuzzy goal programme–based sustainable Greenfield supply network design for tyre retreading industry. Int. J. Adv. Manuf. Technol..

[28] Wang B., Wang Y., Li C., Gao A. (2022). Evolution and Regulation of Radial Structure of PAN Pre-Oxidized Fiber Based on the Fine Denier Model. Materials.

[29] Nakonieczny D.S., Martynková G.S., Hundáková M., Kratošová G., Holešová S., Kupková J., Pazourková L., Majewska J. (2022). Alkali-Treated Alumina and Zirconia Powders Decorated with Hydroxyapatite for Prospective Biomedical Applications. Materials.

[30] Houssat M., Villeneuve-Faure C., Lahoud Dignat N., Locatelli M.-L., Cambronne J.-P. (2021). Temperature Influence on PI/Si3N4 Nanocomposite Dielectric Properties: A Multiscale Approach. Polymers.

[31] Sahu M., Narasimhan L., Raichur A.M., Sover A., Ciobanu R.C., Lucanu N., Aradoaei M. (2021). Improving Fracture Toughness of Tetrafunctional Epoxy with Functionalized 2D Molybdenum Disulfide Nanosheets. Polymers.

[32] Haberstroh E., Michaeli W., Henze E. (2002). Simulation of the Filling and Curing Phase in Injection Molding of Liquid Silicone Rubber (LSR). J. Reinf. Plast. Compos..

[33] Kuo C.-C., Liu H.-A., Lu H.-Y., Shi P.-R. (2022). Development and application of a mold clamping mechanism for improving dimensional accuracy of vacuum casting parts and reducing mold production cost. Int. J. Adv. Manuf. Technol..

[34] Kuo C.-C., Jiang Z.-F., Yang X.-Y., Chu S.-X., Wu J.-Q. (2020). Characterization of a direct metal printed injection mold with different conformal cooling channels. Int. J. Adv. Manuf. Technol..

[35] Martinho P.G., Pouzada A.S. (2021). Alternative materials in moulding elements of hybrid moulds: Structural integrity and tribological aspects. Int. J. Adv. Manuf. Technol..

